# Optimization design of the large area Dynode-MCP-PMT

**DOI:** 10.1038/s41598-022-14671-3

**Published:** 2022-06-21

**Authors:** Lin Chen, Xingchao Wang, Jianli He, Liping Tian, Jinshou Tian, Qilong Wang, Yunji Wang, Jie Yang, Junshuo Qian, Fan Zhang

**Affiliations:** 1 School of Network and Communication Engineering, Jinling Institute of Technology, Nanjing, 211169 China; 2 School of Electronic Science and Engineering, Southeast University, Nanjing, 210096 China; 3 North Night Vision Technology (NNVT) CO., LTD, Nanjing, 210110 China; 4 Inner Mongolia University of Science and Technology, Baotou, 014010 China; 5 State Key Laboratory of Transient Optics and Photonics, Xi’an Institute of Optics and Precision Mechanics (XIOPM), Chinese Academy of Sciences (CAS), Xi’an, 710119 China

**Keywords:** Electronics, photonics and device physics, Nuclear physics

## Abstract

The optimization work of a newly proposed 20-in. photomultiplier tube based on dynode and microchannel plates (Dynode-MCP-PMT) are conducted in this paper. Three-dimensional models are developed in CST STUDIO SUITE to systematically investigate the effects of the size and bias voltage of the two focusing electrodes, dynode and the glass envelop handle based on the Finite Integral Technique and Monte Carlo method. Results predict that the collection efficiency and the transit time spread of the optimized design are substantially improved which are 100% and 3.7 ns.

## Introduction

Large area photomultiplier tubes based on microchannel plates (MCP-PMTs)^1–3^ are widely used in large scale neutrino and cosmic ray experiments. Large photocathode coverage, high quantum efficiency (QE) and collection efficiency (CE, which is defined as the probability that photoelectrons will land on the effective area of the first dynode) are critical parameters for it. Even so, there is a flaw. Owing to the employment of the coated MCPs, a long tail is observed in the time distribution of the output electrons (TDOE), which deteriorates the MCP-PMT time performance.

**Figure 1:**
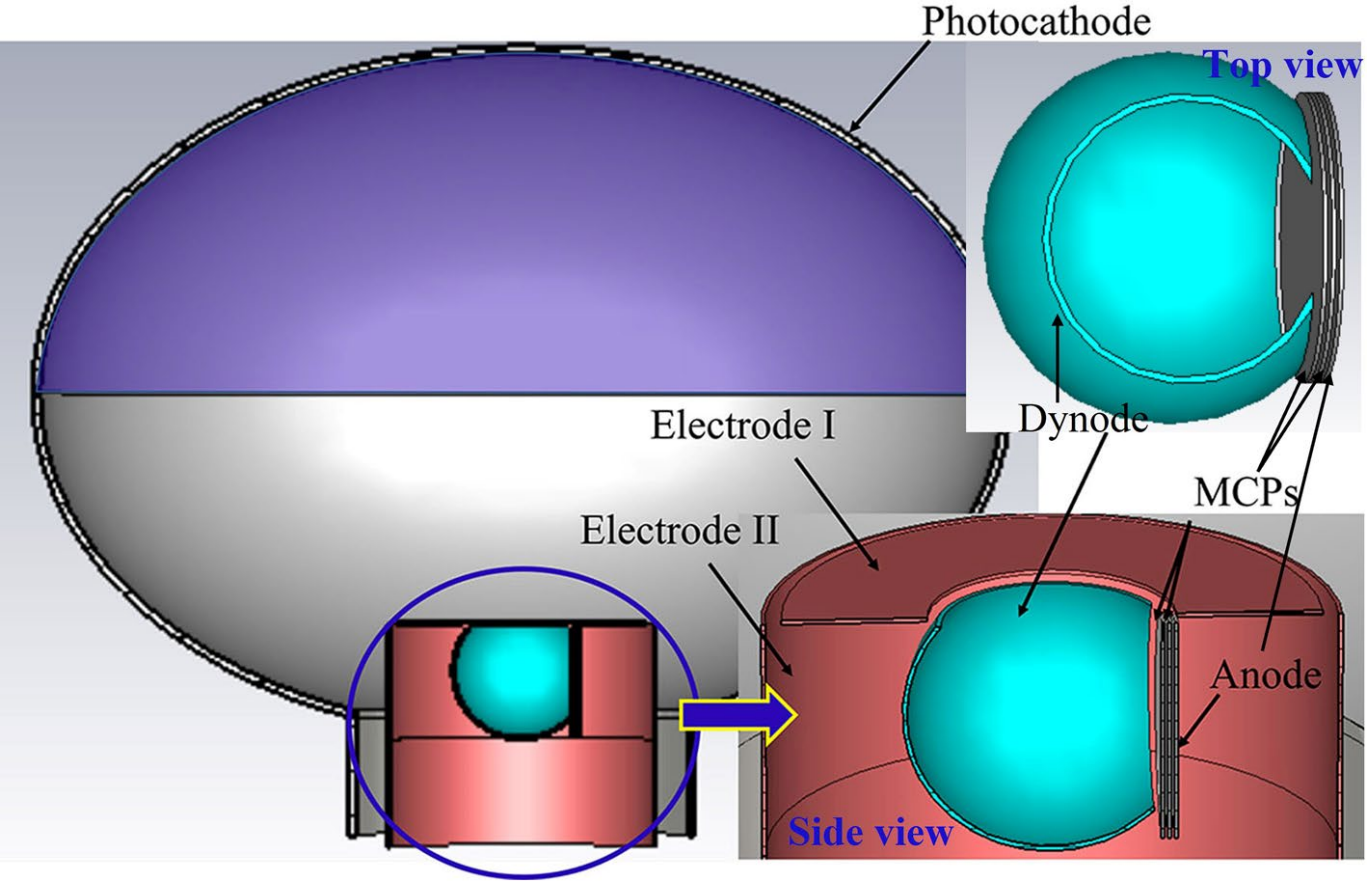
Schematic diagram of the Dynode-MCP-PMT.

To suppress the tail, a novel PMT based on a dynode and a pair of uncoated MCPs (Dynode-MCP-PMT) shown in Fig. 1 is proposed recently. In the Dynode-MCP-PMT, a spherical dynode with two orthogonal openings and a pair of uncoated MCPs are designed as the multiplication system. One of the dynode openings faces to the ellipsoidal glass cavity to collect photoelectrons, the other faces to the MCPs to transport secondaries for further multiplication. Potential applied on the first MCP is higher than that on dynode to generate stronger electric field and attract secondaries. The focusing system includes two electrodes. Electrode I is designed upon the dynode to prevent the electric field generated by MCPs overflowing. Electrode II is a hollowed cylinder to shield the supporting structures and the electrode wires of the multiplication subassembly. Owing to the application of the large opening dynode and the uncoated MCPs, the Dynode-MCP-PMT exhibits outstanding CE performance which is 100%, good transit time spread (TTS, which is defined as the transit time fluctuation of each photoelectron pulse) of 7.2 ns which is pretty good compared to the past observed data (12 ns) in Ref. 2 and 3. Nevertheless, compared to the TTS which less than 5 ns of the traditional dynode PMTs, 7.2 ns is not competitive.

This paper presents the optimization work on the 20-in. Dynode-MCP-PMT aiming to achieve smaller TTS (less than 5 ns) and high CE. Effects of the bias voltage and the size for several components on the electron collection process are systematically investigated.

## Theory and simulation details

Simulations are conducted to systemically study the performances including CE and transit time distribution by the 20-in. Dynode-MCP-PMT models.

CST Studio Suite^4^ is adopted to build the model and calculate the electric fields, electron trajectories, energies and velocities based on the Finite Integral Technique and Monte Carlo method. The feasibility and effectiveness of this simulation approach has already been validated by previous study^5–7^. Particularly, a good agreement was found between the experiments and simulation results in Ref.^7^ which adopt the same simulation method and the similar model as ours. Photoelectrons impacting on the dynode will excite secondaries. Inspired from previous researches^8–14^, present simulation employs the Furman secondary electron emission model^15^. Three components of secondaris are well simulated including backscattered electrons, rediffused electrons and true-secondary electrons.

Dependence of CE and transit time distribution on the bias voltage and size of the two focusing electrodes, dynode and glass envelop handle is systematically investigated. In the following simulations, photoelectrons are emitted from the entire top hemisphere. Photocathode is applied with 0 V. Potential difference between the photocathode and the first MCP-in is 2000 V. Only one parameter is varied at a time, while the others are kept constant, with values listed in Table 1. Owing to the short distance and high potential difference between the dynode and anode, the electron transit time between them is around several hundred picoseconds and TTS is just tens of picoseconds which thus are negligible. Therefore, optimizing the photocathode to the dynode electron optics system is the focal point in this paper. CE and TTS are evaluated by a 2D monitor which records the electron transit time and position information.

**Table 1:** Parameters of the 20-in. Dynode-MCP-PMT prototype.

Item	Parameter	Prototype
Glass envelop	Major axis/mm	ϕ 508
	Minor axis/mm	ϕ 360
	Handle diameter (D_h_)/mm	ϕ 180
Electrode I	Outer diameter (D_I-out_)/mm	ϕ 130
	Inner diameter (D_I-in_)/mm	ϕ 60
	Bias voltage (U_I_)/V	0
Electrode II	diameter (D_II_)/mm	ϕ 140
	Bias voltage (U_II_)/V	1500
Dynode	diameter (D_d_)/mm	ϕ 70
	Bias voltage (U_d_)/V	1000

## Optimization design and results analysis

### Electrode I

**Figure 2:**
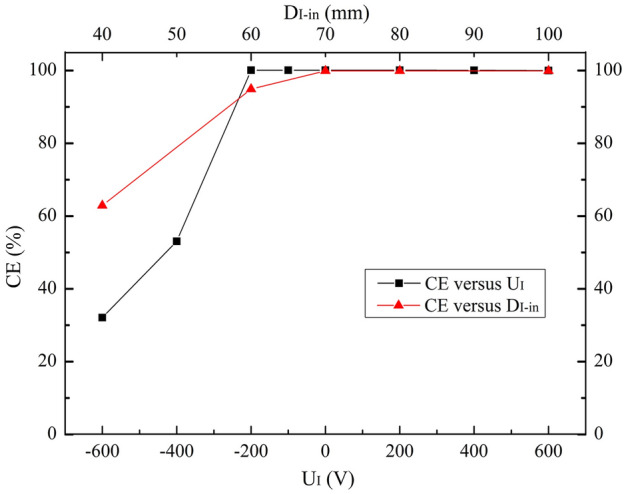
CE versus U_I_ and D_I-in_ over the ranges of − 600 V ≤ U_I_ ≤ 600 V and 40 mm ≤ D_I-in_ ≤ 100 mm.

**Figure 3:**
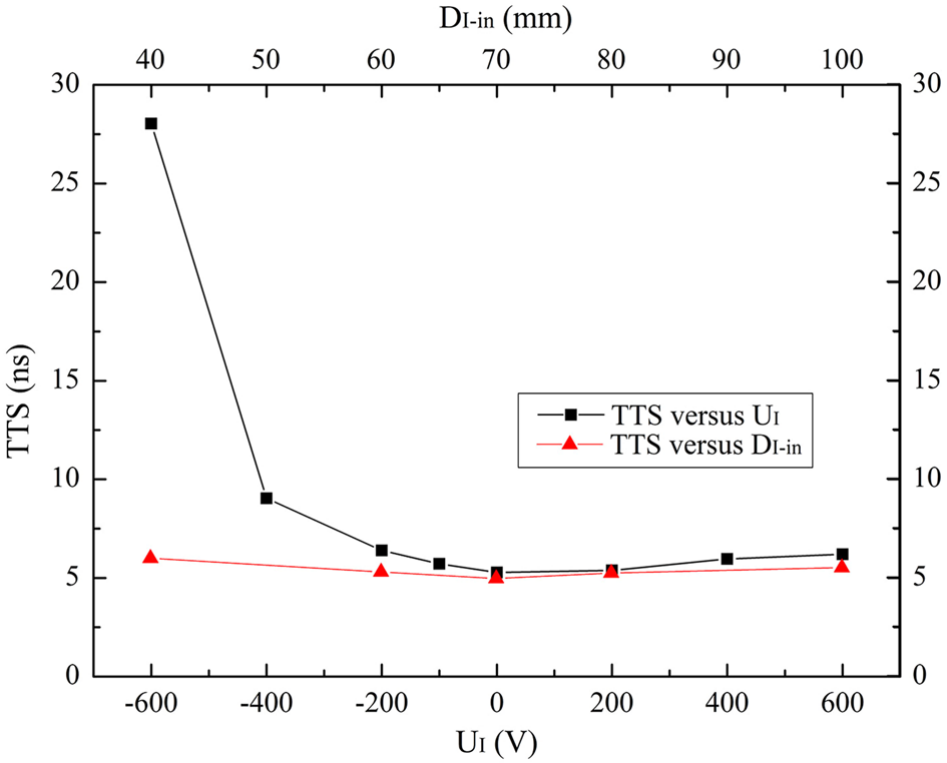
TTS versus U_I_ and D_I-in_ over the ranges of − 600 V ≤ U_I_ ≤ 600 V and 40 mm ≤ D_I-in_ ≤ 100 mm.

The dependence of CE and TTS on the applied voltage (U_I_) and the inner diameter (D_I-in_) of the electrode I is well investigated. U_I_ and D_I-in_ are varied from − 600 V to 600 V and 40 mm to 100 mm. Results are graphically represented in Fig. 2 and Fig. 3.

As can be seen in Fig. 2, the tendencies of CE vs. U_I_ and D_I-in_ are similar. With the increases of U_I_ and D_I-in_, CEs gradually increase until the maximum of 100% at U_I_ = − 200 V and D_I-in_ = 70 mm and then remain constant. Obviously, negative voltage weakens the electric field upon the dynode, which repels a portion of photoelectrons and makes them be attracted by the electrode II. Owing to the 0 V bias voltage, small D_I-in_ not only weakens the electric field upon the dynode, but also interrupt the collection process, which results in the low CE.

TTS vs. U_I_ also has the similar change trend as TTS vs. D_I-in_. With the increasing U_I_ and D_I-in_, TTSs gradually decrease until the minimum and then increase slightly, whose reason can be ascribed. As mentioned above, low U_I_ generates weak electric field in which TTS is more affected by the initial momentum of photoelectrons. On the contrary, high U_I_ generates strong field upon the dynode, which enlarges the speed difference between the photoelectrons from the top and other areas of the photocathode, and thus widens TTS. Results show that at U_I_ = 0 V, TTS reaches the minimum value of 5.3 ns. Similarly, D_I-in_ determines the field strength in the PMT and finally affects TTS. The minimum TTS is 4.9 ns at D_I-in_ = 70 mm.

Based on the consideration of both high CE and short TTS, U_I_ = 0 V and D_I-in_ = 70 mm are studied out as the optimized values of electrode I, assuming that other parameter values are fixed as listed in Table 1.

### Electrode II

**Figure 4:**
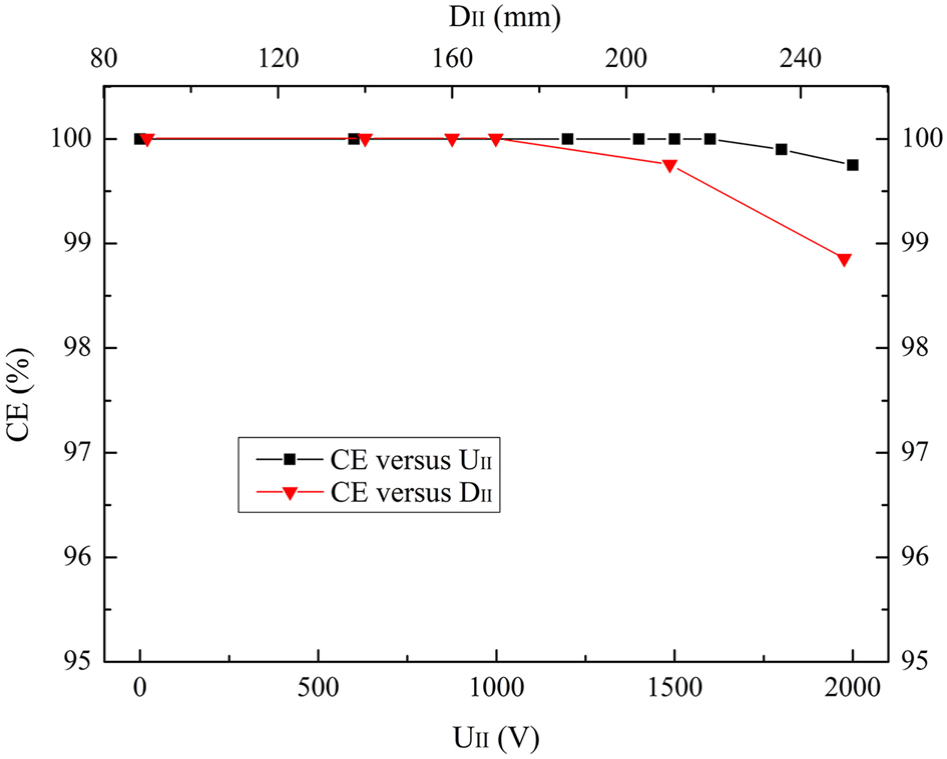
CE versus U_II_ and D_II_ over the ranges of 0 V ≤ U_II_ ≤ 2000 V and 90 mm ≤ D_II_ ≤ 250 mm.

**Figure 5:**
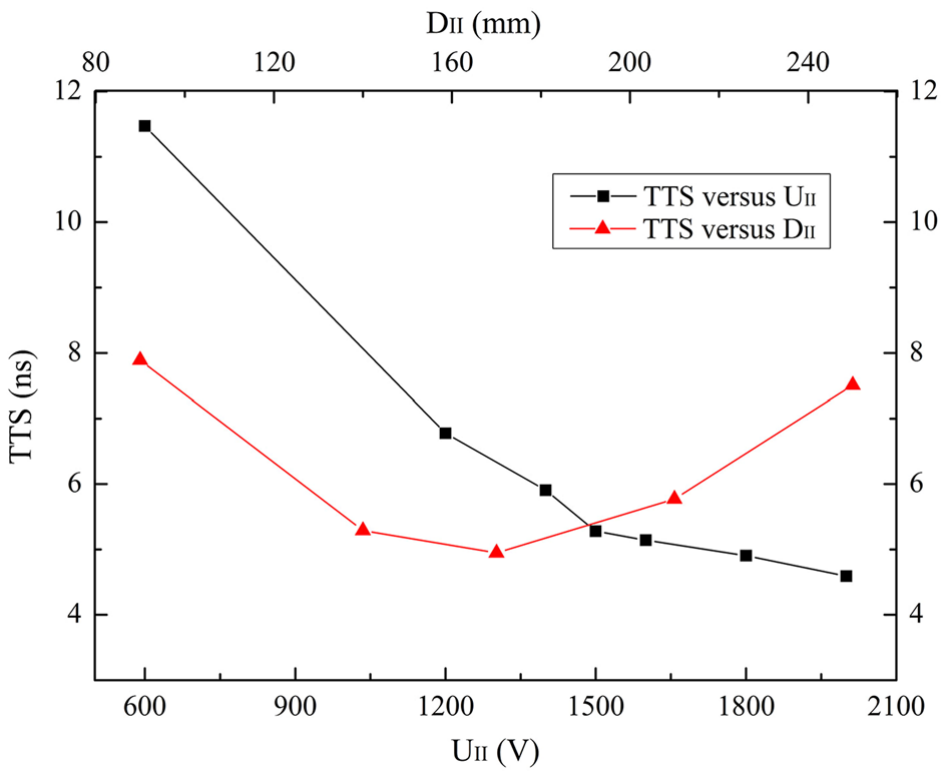
TTS versus U_II_ and D_II_ over the ranges of 0 V ≤ U_II_ ≤ 2000 V and 90 mm ≤ D_II_ ≤ 250 mm.

CE and TTS performances for the bias voltage (U_II_) and diameter (D_II_) of the electrode II over the ranges of 0 V ≤ U_II_ ≤ 2000 V and 90 mm ≤ D_II_ ≤ 250 mm are studied. Results are exhibited in Fig. 4 and Fig. 5.

Two similar tendencies are observed in Fig. 4. CEs keep 100% until U_II_ = 1600 V and D_II_ = 170 mm, then decrease slightly. It can be seen form the electron trajectories that with the increase of U_II_ and D_II_, more photoelectrons tend to be attracted by the electrode II, which deteriorates CE.

A declining TTS is observed for increasing U_II_ in Fig. 5. At U_II_ = 2000 V, TTS is the minimum which is 4.6 ns. With the increase of D_II_, TTS gradually decreases until the minimum of 4.9 ns at D_II_ = 170 mm and then increases. As analyzed in the U_I_ part, D_II_ affects the electric field intensity in the PMT, and thus TTS.

Considering both high CE and short TTS, U_II_ = 1600 V and D_II_ = 170 mm are supposed to be the optimized values, assuming that other parameter values are fixed as listed in Table 1.

### Dynode

Diameter of the dynode (D_d_) affects the electric field distribution in and upon the dynode. The dependence of CE and TTS on the bias voltage of the dynode (U_d_) and D_d_ is systematically investigated over the ranges of 0 V ≤ U_d_ ≤ 2000 V and 70 mm ≤ D_d_ ≤ 130 mm.

**Figure 6:**
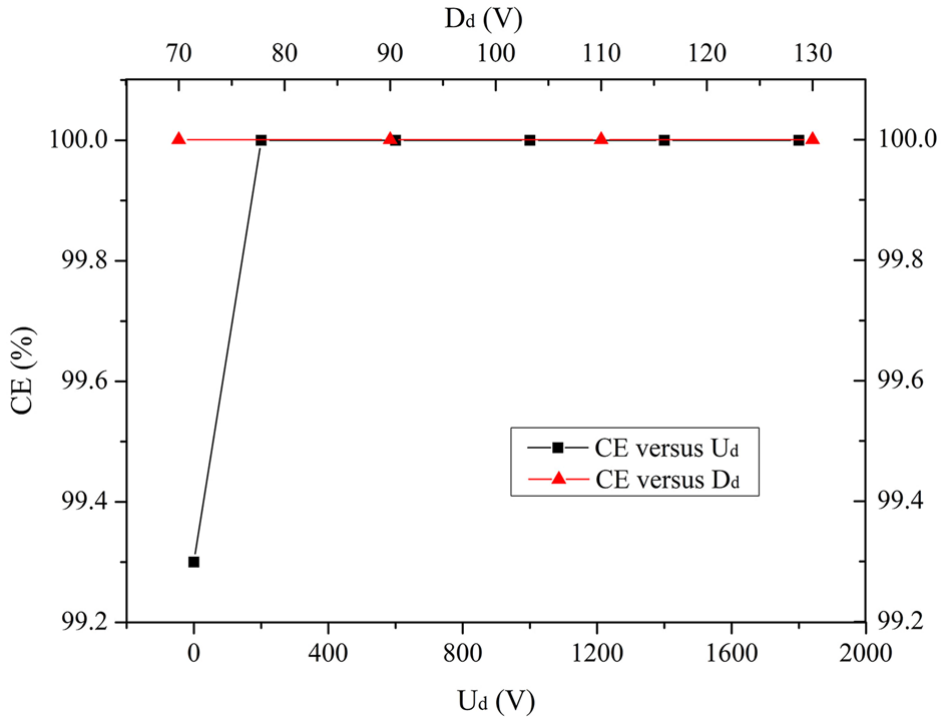
CE versus U_d_ and D_d_ over the ranges of 0 V ≤ U_d_ ≤ 2000 V and 70 mm ≤ D_d_ ≤ 130 mm.

Results in Fig. 6 show that D_d_ has no significant effect on CE. CE remains 100% in the whole interval 0 mm ≤ D_d_ ≤ 2000 mm. CE stayed at 100% for various U_d_ except 0 V which is 99.2%.

**Figure 7:**
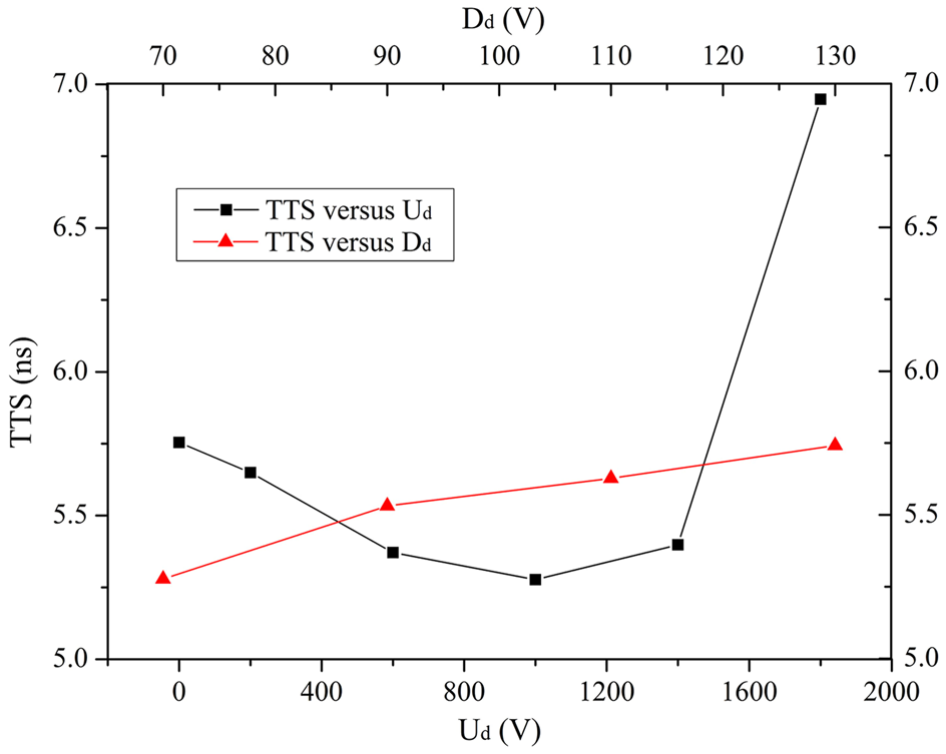
TTS versus U_d_ and D_d_ over the ranges of 0 V ≤ U_d_ ≤ 2000 V and 70 mm ≤ D_d_ ≤ 130 mm.

It is shown in Fig. 7 that TTS gradually decreases to a minimum of 5.3 ns at U_d_ = 1000 V and then increases to some extent with increasing U_d_. The reason is the same as U_I_’s as mentioned above. In addition, D_d_ has the similar effects on TTS as D_I-in_ ≥ 70 mm. An increasing TTS (the minimum is 5.3 ns) is observed for the increasing D_d_.

Based on above discussion, U_d_ = 1000 V and D_d_ = 70 mm are employed as the optimized values for the dynode, assuming that other parameter values are fixed as listed in Table 1.

### Glass envelop handle

The inner surfaces of the bottom hemisphere and the handle of the glass envelope are coated with the aluminum thin layer which is electrically connected with the cathode (0 V). Diameter of the glass envelope handle (D_h_) impacts the electric field distribution, and thus the time properties and CE. Effects of D_h_ on CE and TTS are studied in the interval of 180 mm ≤ D_h_ ≤ 340 mm.

**Figure 8:**
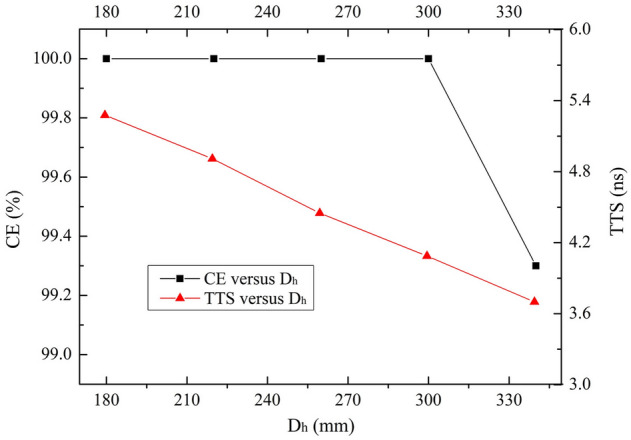
CE and TTS as functions of D_h_ over the range of 180 mm ≤ D_h_ ≤ 340 mm.

As exhibited in Fig. 8 that CE remains 100% first and then decreases after 300 mm. The reason is similar as D_II_’s. Besides, a decreasing TTS is observed. The electric field shielded by the glass handle is gradually released with the increase of D_h_, which enhances the electric field in the PMT. The strong field reduces the momentum difference of photoelectrons and narrows TTS. Therefore, D_h_ should be optimized into 300 mm.

### The optimized design

Inspired by above simulations, a set geometry and operating parameters of the Dynode-MCP-PMT are proposed for better performance as summarized in Table 2.

**Table 2:** Parameters of the optimized 20-in. Dynode-MCP-PMT.

Item	Parameter	Optimized design
Glass envelop	Major axis/mm	ϕ 508
	Minor axis/mm	ϕ 360
	Handle diameter (D_h_)/mm	ϕ 300
Electrode I	Outer diameter (D_I-out_)/mm	ϕ 360
	Inner diameter (D_I-in_)/mm	ϕ 70
	Bias voltage (U_I_)/V	0
Electrode II	diameter (D_II_)/mm	ϕ 170
	Bias voltage (U_II_)/V	1600
Dynode	diameter (D_d_)/mm	ϕ 70
	Bias voltage (U_d_)/V	1000

Results show that CE of the optimized model is 100%. TTS is 3.7 ns which is less than 5 ns and almost cut the 7.2 ns (before optimization) in half. Besides, the gain of the first dynode is 6.4 which is benefit for the total gain.”

## Discussion

The optimization work of the Dynode-MCP-PMT is conducted in this work. The performances of the PMT for a wide range of operating and geometry conditions are systematically investigated. Results show that the optimized CE and TTS are 100% and 3.7 ns at U_I_ = 0 V, D_I-in_ = 70 mm, U_II_ = 1600 V, D_II_ = 170 mm, U_d_ = 1000 V, D_d_ = 70 mm and D_h_ = 300 mm, which are superior to those of the nonoptimized one. The optimization approach will be used as significant guidelines for the development of high-performance PMT.
